# Doxycycline post-exposure prophylaxis (doxyPEP): balancing promise and prudence in the prevention of sexually transmitted infections

**DOI:** 10.2807/1560-7917.ES.2025.30.26.2500454

**Published:** 2025-07-03

**Authors:** Fiona Lyons,, Adam Shanley

**Affiliations:** 1 Department of Genitourinary Medicine and Infectious Diseases, St. James’s Hospital, Dublin, Ireland; 2 Sexual Health Programme, Health Service Executive, Dublin, Ireland; 3 School of Medicine, Trinity College Dublin, Dublin, Ireland

**Keywords:** sexually transmitted infections, doxycycline, prophylaxis, doxyPEP

Epidemiological data suggest that the World Health Organization (WHO) 2023 targets for reductions in sexually transmitted infections (STIs) will not be met [1,2]. The clinical utility of doxycycline as post-exposure prophylaxis (doxyPEP) in averting incident STIs is evident, particularly in populations at high risk [3-6]. However, questions and concerns remain regarding the impact on antimicrobial resistance (AMR), the composition of the human microbiome, and antimicrobial consumption. Some countries have developed guidelines recommending doxyPEP use in certain circumstance [7,8], while others have issued more cautious or restrictive position statements [9-12]. Yet, most countries in Europe have not articulated a stance, potentially awaiting guidance from international public health bodies such as the WHO and European Centre for Disease Prevention and Control (ECDC), which have already convened expert advisory groups [13,14].

Despite this, informal use of doxyPEP i.e. self-sourcing doxyPEP without prescription or sourcing a prescription for a different use of doxycycline, is already occurring and intention to use continues to rise independently of formal policy or guidelines. In their paper in this issue of *Eurosurveillance*, Teker et al., demonstrate growing awareness, use, and intention to use among key populations [15]. Their findings from an online survey among men who have sex with men (MSM) and transgender and gender diverse people in the Netherlands, offer important insights into user motivations, knowledge gaps, and the influence of community and peer networks in shaping health behaviours.

The efficacy of doxyPEP in reducing STIs is compelling for syphilis, given recent increasing rates of infection in key populations in Europe [1] and the severe complications it can cause. Trials also consistently showed a marked reduction in incidence of chlamydia with doxyPEP [3-5]. Regarding gonorrhoea, the results have been mixed. While the United States doxyPEP study observed some reduction in gonorrhoea incidence, this was less pronounced than for syphilis and chlamydia [4]. The variability was attributed in part to pre-existing tetracycline resistance in *Neisseria gonorrhoeae*, which is endemic in some populations.

The utility of asymptomatic chlamydia (and gonorrhoea) testing in gay, bisexual and other (gb)MSM in connection with the use of HIV pre-exposure prophylaxis is increasingly debated [16,17]. Some countries have already reduced asymptomatic testing [18,19]. Notwithstanding potential changes to the approach to asymptomatic testing, there may be some individuals who experience frequent symptomatic *Chlamydia trachomatis* infections, including lymphogranuloma venereum, for whom doxyPEP may be an important intervention.

Aside from reductions in syphilis and chlamydia, doxyPEP can decrease anxiety around sex, and this can result in a broader sense of sexual empowerment [20]. Good sexual health should not be solely defined by the absence of STIs. It should also encompass the reduction of related shame, anxiety, and stigma, as well as the enhancement of pleasure as core to sexual health. It is imperative that there is open, complete dialogue with current and potential doxyPEP users which is not limited to potential risks and unknowns.

DoxyPEP use has prompted substantial concern from health professionals regarding its potential to contribute to AMR. Increases have been observed in tetracycline-resistant gonococcal strains among doxyPEP users [4]. *Chlamydia trachomatis* and *Treponema pallidum* currently show low levels of resistance to tetracyclines, but sustained selective antimicrobial pressure could change that. Beyond STIs, the off-target selection pressure exerted by doxycycline may promote resistance in commensal organisms. This poses risks not just for individual patients but also for population-level antibiotic effectiveness, including in future treatment of respiratory, urinary, and skin infections, and warrants robust monitoring and surveillance.

Antimicrobial resistance is rightly a global public health concern, and addressing it requires sustained attention across multiple sectors. While significant progress has been made in addressing overuse, antimicrobial use remains substantial in both human and agricultural sectors. In this context, interventions like doxyPEP, which are targeted, evidence-based, and focused on populations experiencing a high burden of STIs, warrant careful consideration and public health stewardship. Rather than framing such strategies as contributing to resistance in isolation, it is important to situate them within the wider AMR landscape to ensure research and policy responses are appropriately balanced and effective.

Doxycycline, while generally well-tolerated, is a broad-spectrum antibiotic that can alter the human microbiome. Long-term or frequent use may disrupt the balance of gastrointestinal, oral, and skin flora, potentially increasing the risk of opportunistic infections such as *Clostridioides difficile* or yeast overgrowth, and contribute to dysbiosis-related health issues. This concern is important when considering doxyPEP as a preventive measure that may be used frequently (e.g. multiple times per month) by sexually active individuals. While trial participants have not reported major adverse events related to microbiome changes [5], longitudinal data are lacking. Given the growing recognition of the microbiome’s role in immune modulation and chronic disease risk, further research is essential.

Online fora, community-based organisations, and sexual health clinics report growing awareness and use of antibiotics after condomless sex, often without medical supervision [21,22]. The article from Teker et al. increases awareness of informal use among MSM and trans communities in a high-income setting. There was a substantial intention to use doxyPEP if formally recommended, particularly among those with a history of recurrent STIs or multiple partners. This underscores both the perceived need for additional STI prevention tools and the potential risk of unregulated use that could exacerbate resistance and adverse effects. Encouragingly, many individuals in the study reported using or intending to use doxyPEP/pre-exposure prophylaxis as a proactive measure to safeguard their health and support their sexual wellbeing. Their motivations included a strong desire to protect themselves and their partners, reduce the transmission of STIs within their communities, and alleviate the anxiety and stigma often associated with STIs. These motivations reflect a pattern observed in other studies [20,23,24], highlighting how doxyPEP use is often driven by care, responsibility, and a commitment to informed sexual health. This sense of shared responsibility reflects strong community values around prevention and wellbeing, which should be leveraged. The high intention to use doxyPEP in this study is shaped by peer influence, community discussion, and the presence of trusted resources. This is an important reminder that accurate, culturally relevant messaging co-developed by community and healthcare/public health professionals can support people in making safer and more informed decisions, regardless of whether national guidelines are in place.

The study findings suggest that those most likely to benefit from doxyPEP, have appropriately selected themselves. Participants who reported recent use were more likely to be living with HIV or using oral HIV PrEP, had a history of bacterial STIs, and more frequently reported sexual practices that increase STI risk. The authors take an important step towards inclusivity as this is one of very few studies to include participants beyond cisgender gbMSM. Understanding which communities need and want to use doxyPEP is important in addressing inequities. Without an evidence base that is representative of ciswomen and transgender men, disparities are being built in from the outset. Notwithstanding the inclusive nature of the study, the authors’ suggestion that implementation strategies focus on small, high-incidence sub-populations to limit antibiotic consumption, may risk entrenching inequities and overlooking those who are choosing to use doxyPEP anyway. Restricting access without offering pathways to care does not prevent use, it makes it harder for people to do so safely and with appropriate oversight. Engaging doxyPEP users and those intending to use it provides them with information to make informed choices and become regular care participants. This not only offers opportunities for monitoring but also builds trust. This trust will be vital if, in the future, there is a need for de-implementation or behaviour change.

While the majority of respondents in the current study used doxycycline, almost one in four used other antibiotics. This, alongside the fact that a notable proportion of individuals used doxycycline before an exposure, further illustrates the importance of engaging with the gbMSM and trans communities in articulating clearly what is known about doxyPEP, including correct dosing, correct antibiotic choice alongside a clear and open articulation of the potential for AMR and the unknown implications on the microbiome.

As doxyPEP is already happening, public health experts and clinicians in the field of STIs have a collective responsibility to understand its extent and should not miss opportunities to engage with individuals who are using it or intending to do so. Surveillance and monitoring are necessary to assess the public health impact of doxyPEP use. This includes tracking trends in STI incidence, AMR patterns, and access to doxyPEP across different populations. Reliable data will help inform evidence-based guidelines and detect unintended consequences early. Robust systems for monitoring also allow for the adjustment of strategies as new evidence emerges, ensuring that responses are agile, equitable, and sustainable in the long term.

Collaboration among healthcare providers, researchers, public health agencies, and community organisations is essential. Collaborative approaches have worked successfully in the past, as recently demonstrated by coordinated responses to the 2022/23 mpox outbreak, for example in Ireland [25]. Through interdisciplinary partnerships, we can ensure that clinical research aligns with community priorities, that messaging is culturally appropriate and medically accurate, and that policy decisions are grounded in both science and lived experience.

## Note

Fiona Lyons is a consultant in Genitourinary Medicine at St James’s Hospital in Dublin, Medical Director and Clinical Lead of the HSE Sexual Health Programme and a Clinical Professor at the School of Medicine, Trinity College Dublin.

Adam Shanley is a doctoral researcher in health policy and management at Trinity College Dublin and champion for community-led public health.
